# Recyclable NiO/sepiolite as adsorbent to remove organic dye and its regeneration

**DOI:** 10.1038/s41598-022-06849-6

**Published:** 2022-02-21

**Authors:** Shu Gao, Dahua Wang, Zhi Huang, Chengyuan Su, Menglin Chen, Xiangfeng Lin

**Affiliations:** 1 Key Laboratory of Ecology of Rare and Endangered Species and Environmental Protection, Guangxi Normal University, Ministry of Education, Guilin, 541004 People’s Republic of China; 2 School of Environment and Resource, Guangxi Normal University, Guilin, 541004 People’s Republic of China; 3 University Key Laboratory of Karst Ecology and Environmental Change of Guangxi Province, Guangxi Normal University, Guilin, 541004 People’s Republic of China

**Keywords:** Environmental sciences, Chemistry, Materials science

## Abstract

In this study, the impregnation synthesis of NiO/sepiolite and its application for dye removal during wastewater treatment is introduced. The NiO/sepiolite materials act as an adsorbent/catalyst. It comprises a unique combination of adsorption and high-temperature gas flow regeneration (the NiO/sepiolite acts as a catalyst at this stage, using regeneration rate as evaluation index of catalytic activity of NiO/sepiolite) in a single unit, in which the NiO/sepiolite was regenerated and reused for the next round adsorption of dye. An aqueous solution of methylene blue was used to evaluate the adsorption and regeneration performance of the adsorbent/catalyst. The regeneration rate reached 74% when the reaction time and temperature were 7 min and 350 °C, respectively. The effects of the regeneration temperature and volume fraction of O_2_ on the regeneration rate were investigated. And the regeneration reaction kinetics was provided. The combination of adsorptive and catalytic properties in the NiO/sepiolite composites received interesting results for removing refractory biodegradable organic pollutants. This work provides new insights for the removal of dye from wastewater using Ni catalysts supported on natural low-cost clay.

## Introduction

Synthetic dyes are a type of hazardous and toxic pollutant, and most of them are derived from the printing and dye industries. If not treated appropriately, dyes will cause damage to the environment and human beings. Hence, the removal of dye from waste effluents is environmentally important. Various methods, such as catalytic wet oxidation, coagulation, ultrafiltration, ozonation, sedimentation, reverse osmosis, flotation, precipitation, photodegradation, adsorption, biological processes, and others have been successfully applied for dye removal. Among these methods, improvements in the degradation efficiency, as well as reductions in the cost of organic substance removal^1–4^, are concerns.

The removal of pollutants from water by adsorption is one of the most promising techniques due to its operational convenience and low cost when applied to current water treatment processes^1,5^. Adsorption using activated carbons is highly attractive for removing toxic and refractory biodegradable organic contaminants^6^. However, this approach also has some shortcomings. For instance, it causes environmental problems when the activated carbons are exhausted and disposed of afterward in landfills or by incineration^7^. Therefore, it is important to regenerate the adsorbents and reuse them. Much attention has been paid to alternative regeneration techniques in recent years. These techniques include microbial regeneration^8^, wet oxidation processes^9^, chemical regeneration^10–12^, solvent regeneration^13^, microwave-assisted regeneration^14^, electrochemical regeneration^6,15,16^, solar regeneration^17^, and thermal regeneration^18^. Clearly, the above reports have strongly demonstrated general and very effective strategies to improve the regenerative performance. Among these regeneration techniques, thermal regeneration is effective for the reuse of adsorbents. In this study, it is shown that high-temperature gas flow regeneration processes are a promising option, since they are fast processes that are performed in situ within the adsorption column under ambient conditions and at lower temperatures. Our previous report demonstrated that high-temperature gas flow regeneration was an effective process^19^. This technique includes adsorption and high-temperature gas flow regeneration that takes place within a single unit. High-temperature gas flow regeneration demonstrates several potential advantages compared to thermal regeneration. (1) Shorter regeneration time. The regeneration rate can reach 75% after 7 min of reaction time under a high-temperature gas flow, while thermal regeneration may require much more time^20^, and (2) lower energy consumption.

Adsorption on a low-cost adsorbent is an attractive technology for water treatment^21^. Clay minerals, such as montmorillonite, vermiculite, and sepiolite, are of great interest in the study of adsorption for removing pollutants and as supporting materials for catalysts due to their peculiar physicochemical properties, as well as their abundance in nature, low costs, and environmentally friendliness. Among the clay mineral family, sepiolite (Mg_8_Si_12_O_30_(OH)_4_(OH_2_)_4_·8H_2_O), with channels of molecular dimensions, determines that it may be considered an adsorbent with a uniform microporosity and external porosity^22,23^. These qualities make sepiolite have a huge specific surface area and high porosity, which can provide more reaction sites for catalytic activity and prevent nano-catalysts from aggregation^24,25^. Therefore, sepiolite has been employed as an adsorbent or support and binder for composites with metal oxides^26^. When catalysts are loaded on sepiolite, their catalytic activity, recyclability, and flexibility are further enhanced due to the unique structure and morphology of the sepiolite^27^.

The adsorption capacity that is restricted to an external surface can be enhanced using thermal or chemical modification, which are common methods for modifying clay minerals^5^. This modification can remove some impurities and increase the adsorption capacity of clay. Highly dispersed metal or metal oxide particles on a sepiolite support have shown improved catalytic activity in catalyzed processes, such as core–shell Ag@Pt^27^, coprous oxide^28^, iron^29^, nickel^30^, vanadium^31^, and Ti^32^ oxides. Among these metal oxides, Ag and Pt are noble metals that are restricted in application due to their high costs. Cuprous oxide/sepiolite can obtain an organic pollutant degradation rate of 87% under visible light, but the degradation time required is 5 h. Ni is a group VIII metal, and Ni-based catalysts have received extensive attention due to their low costs and high activities in catalytic reactions. In addition, nickel oxide (NiO) particles are very popular in coatings for the catalyst because of their high specific capacitance, inherent environmental friendliness, and practical accessibility^33^. Recently, natural sepiolite promoted with Ni has been used for the sustainable production of hydrogen and in the steam reformation of furfural and toluene. It has exhibited more enhanced catalytic performance than traditional Ni supports, such as SiO_2_, MgO, or Al_2_O_3_^34–36^.

In this study, NiO/sepiolite materials with high adsorption and catalytic activities are synthesized via an impregnation route. The as-prepared samples are then characterized using X-ray diffractometry (XRD), scanning electron microscopy (SEM), Brunauer–Emmet–Teller (BET), thermogravimetric analysis (TGA). The adsorption of dye on the NiO/sepiolite and the subsequent high-temperature gas flow regeneration behavior are studied. In a single adsorption-regeneration unit, the NiO/sepiolite materials demonstrated excellent adsorption properties, as well as catalytic activities and thermal stabilities. In addition, the kinetics of the catalytic degradation of methylene blue over NiO/sepiolite is discussed. The regeneration temperature for the NiO/sepiolite materials was much lower than in our previous work^19^.

## Experiment

### Preparation of the NiO/sepiolite adsorbents/catalysts

The natural sepiolite was purchased from Henan Province (China). All of the chemical reagents were of analytical grade and used as received without further purification. Deionized water was used for all of the experiments. Prior to use, the raw sepiolite was pre-treated and then placed into deionized water and stirred for 24 h. Then, the sepiolite was filtered and rinsed three times using deionized water and dried at 105 °C for 48 h. Finally, the dried sepiolite was crushed. The NiO/sepiolite adsorbents/catalysts were prepared using impregnation. A typical synthesis procedure for the NiO/sepiolite was as follows: sepiolite with 40–60 mesh was added to a 0.2 mol L^−1^ nickel nitrate solution and impregnated for 24 h. The ratio of solid (sepiolite) to liquid (nickel nitrate solution) was 1:10. Then the solution was removed using filtration. The resulting granulated material was dried in an oven and subsequently calcined under an O_2_ flow at 350 °C for 1 h to obtain the NiO/sepiolite.

### Characterization

A D8-Advance Bruker X-ray diffraction (XRD) powder diffractometer was used to study the crystalline phases of the NiO/sepiolite, in which Cu Kαradiation was used. The chemical compositions of sepiolite and NiO/sepiolite were analysed by X-ray fluorescence (XRF, Rigaku XRF primus-2). A simultaneous differential thermal analysis/thermogravimetric (DTA/TG) system (Perkin-Elmer Pyris Diamond DTA/TG) was used for the thermogravimetric (TG) analysis of samples. The experiments were conducted at temperatures ranging from room temperature up to 1000 °C in O_2_ gas. The specific surface area and pore structure of the NiO/sepiolite were obtained by determining nitrogen adsorption–desorption isotherms at 77 K on a Micromeritics ASAP 2020 apparatus. The morphologies of the samples were examined using a field emission scanning electron microscopy (FE-SEM, FEI Quanta 200 FEG).

### Batch equilibrium adsorption experiments

Batch equilibrium adsorption experiments of a dye on the NiO/sepiolite were conducted. A known mass (1 g) of the NiO/sepiolite with 0.2 L of a methylene blue solution was stirred on a constant temperature shaker at 170 rpm. The concentrations of the dye were in the range of 50–350 mg L^−1^. Samples were gathered at regular intervals and centrifugated, then the concentrations of the dye in the collected samples were analyzed using UV/Vis spectroscopy.

### High-temperature gas flow regeneration

The regeneration of sepiolite and the NiO/sepiolite was conducted in an adsorption column with an inner diameter of 22 mm and a height of 400 mm. A temperature control meter was used to control the regeneration temperature. One round of regeneration included the following procedures:

(i) Initial adsorption: A certain mass of sepiolite or NiO/sepiolite was added to the adsorption column, and a solution of methylene blue was pumped into the adsorption column using a peristaltic pump until the effluent concentration was greater than the penetration point. The initial adsorbent loading qi was then determined.
(ii) High-temperature gas flow regeneration: Once the initial adsorption ended, the regeneration started. Oxygen was used as the oxidation gas to regenerate the NiO/sepiolite adsorbed with methylene blue at the same time that the methylene blue was degraded. The O_2_ gas flow was supplied using an air compressor at the bottom of the adsorption column during regeneration. The regeneration temperature was 350 °C. The catalytic activity of NiO/sepiolite was evaluated by regeneration rate.
(iii) Re-adsorption: A fresh solution of methylene blue was added to the adsorption column to be absorbed by the regenerated sepiolite or NiO/sepiolite. The adsorption process was similar to that of step (i). After adsorption, the regenerated sepiolite or NiO/sepiolite loading qr was determined, at this time, the regeneration rate was calculated. The regeneration rate was calculated by following Eq. (1)^7^:
  1 $${\text{Regeneration}}\;{\text{rate}}\;(\% ) = ({\text{qi/qr}}) \times 100\%$$
  where qr (mg/g) and qi (mg/g) are the equilibrium adsorption capacity of the new and regenerated materials, respectively.

The schematic of adsorption-regeneration cycle system is shown in Schematic 1 (Supplementary Figure), which shows one round of regeneration.

## Results and discussion

### Physico-chemical properties

**Figure 1:**
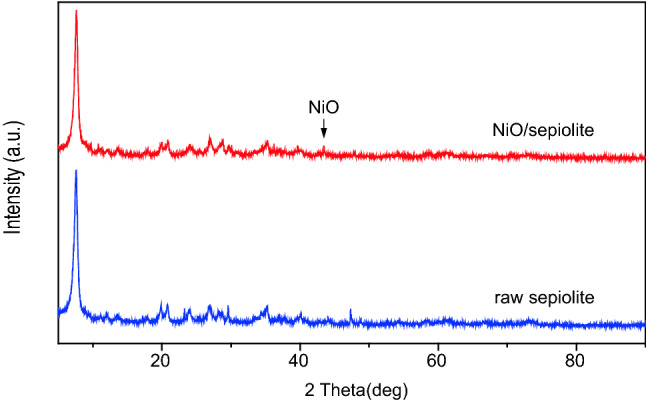
XRD patterns of NiO-sepiolite and raw sepiolite.

The physico-chemical properties of NiO/sepiolite was characterized. X-ray diffractograms for NiO-sepiolite and raw sepiolite are present in Fig. 1. The main diffraction peaks located at 2θ = 7.4°, 20.7°, 23.9°, 26.8°, 28.3°, 35.1°, 40.1° and 47.3°correspond to the characteristic peaks of raw sepiolite. The peak at 2θ = 29.4° is possibly attributed to the characteristic peak of calcium carbonate (JCPDS, No. 86-0174)^27^. The peak located at 2θ = 43.4° belongs to the NiO (JCPDS, No. 73-1519), the signal of 2θ = 37.2° is not visible because of the low content of Ni in the composite. Some relative peaks of sepiolite in NiO/sepiolite is slightly lower than that of the raw one, probably due to the NiO particles deposition. The chemical compositions of sepiolite and NiO/sepiolite are analysed by XRF and the results are listed in Table 1. We can see that the amount load of NiO is 2.1032% from the XRF analysis, which is in agreement with the XRD result that the signal of 2θ = 37.2° is not obvious.

**Table 1:** Chemical composition of sepiolite and NiO/sepiolite (derived from XRF analysis).

Sample	Composition (wt. %)						
	SiO_2_	MgO	CaO	Al_2_O_3_	Fe_2_O_3_	K_2_O	NiO
Sepiolite	55.0505	33.5735	11.1359	0.1749	0.0468	0.0185	**–**
NiO/sepiolite	52.4350	30.8792	14.2931	0.2367	0.0528	–	2.1032

**Figure 2:**
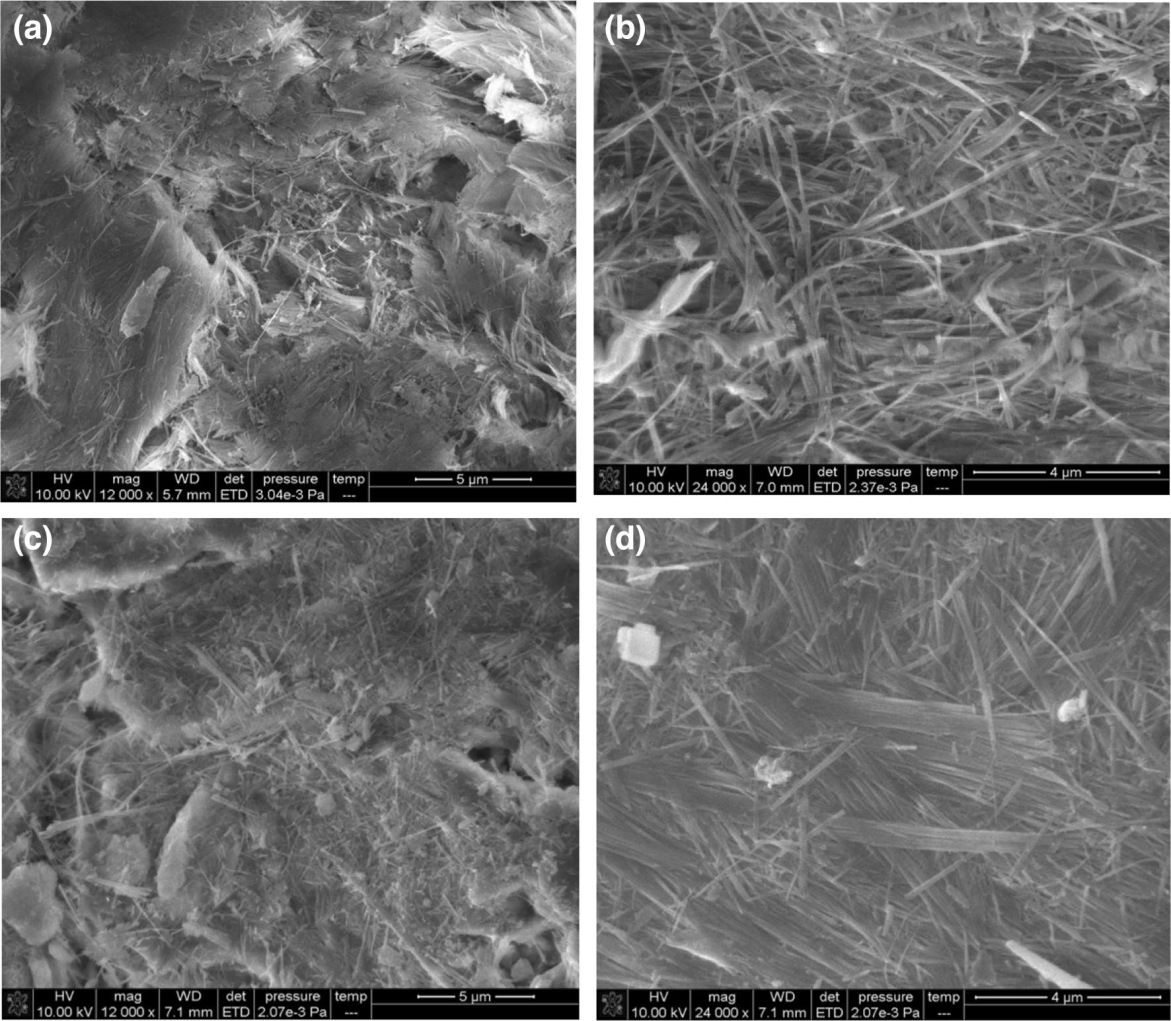
SEM images of sepiolite (**a**), NiO/sepiolite (**b**), regenerated sepiolite (**c**) and regenerated NiO/sepiolite (**d**).

The SEM images of natural sepiolite, NiO/sepiolite, regenerated sepiolite, and regenerated NiO/sepiolite are shown in Fig. 2a–d, respectively. These images show that these samples have fibrous structures. While natural sepiolite (Fig. 2a) agglomerates badly, after regeneration using a high-temperature gas flow, a certain number of cracks were observed. This was likely due to the tension forces generated by the gas flow. Another reason might have been because the process of high-temperature gas flow regeneration is equal to another heat treatment process (Fig. 2c). The fibers of the NiO/sepiolite are longer than those of the natural sepiolite, and some particles aggregated and covered the surface of the fibers (Fig. 2b). The regenerated NiO/sepiolite (Fig. 2d) still maintained the fibrous morphology, although some fibers broke into pieces or granular materials, indicating the modified sepiolite materials had good thermal stability and mechanical strength during high-temperature gas flow regeneration. The results of the SEM detection indicated that the NiO/sepiolite was more stable than sepiolite under tension forces and heat treatment during sample preparation and regeneration.

**Figure 3:**
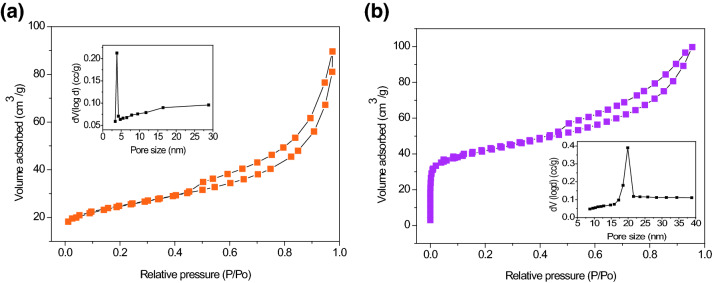
BJH nitrogen adsorption–desorption isotherms of raw sepiolite (**a**), and NiO/sepiolite (**b**), the inset are the corresponding pore size distribution plots.

The BET experiment was performed to confirm the presence of the porous structure in the catalyst and determine the surface area, as well as the pore size distribution. The BET specific surface area of the natural sepiolite and the NiO/sepiolite was calculated to be 87.7 and 150.6 m^2^ g^−1^, respectively, from the nitrogen adsorption isotherm data given in Fig. 3a,b. Both of the N_2_ adsorption–desorption isotherms of the natural sepiolite (Fig. 3a) and the NiO/sepiolite (Fig. 3b) belonged to type IV isotherms with a hysteresis loop. This was due to the presence of the inhomogeneous mesopores. The Barrett–Joyner–Halenda (BJH) pore size distribution curves calculated from the desorption branch are shown in the insets of Fig. 3a,b. The pore size distribution graphs confirmed that the pore size distributions of the two samples were approximately located at 3.8 nm and 19.9 nm, and both of them contained mesopores (20 Å < pore width < 500 Å). It was demonstrated that the loading of NiO affected the sepiolite structure, and both S_BET_ and the pore size increased significantly. A larger specific surface area can provide much more active sites and adsorption interfaces for the adsorption and catalytic degradation of pollutants^27^.

**Figure 4:**
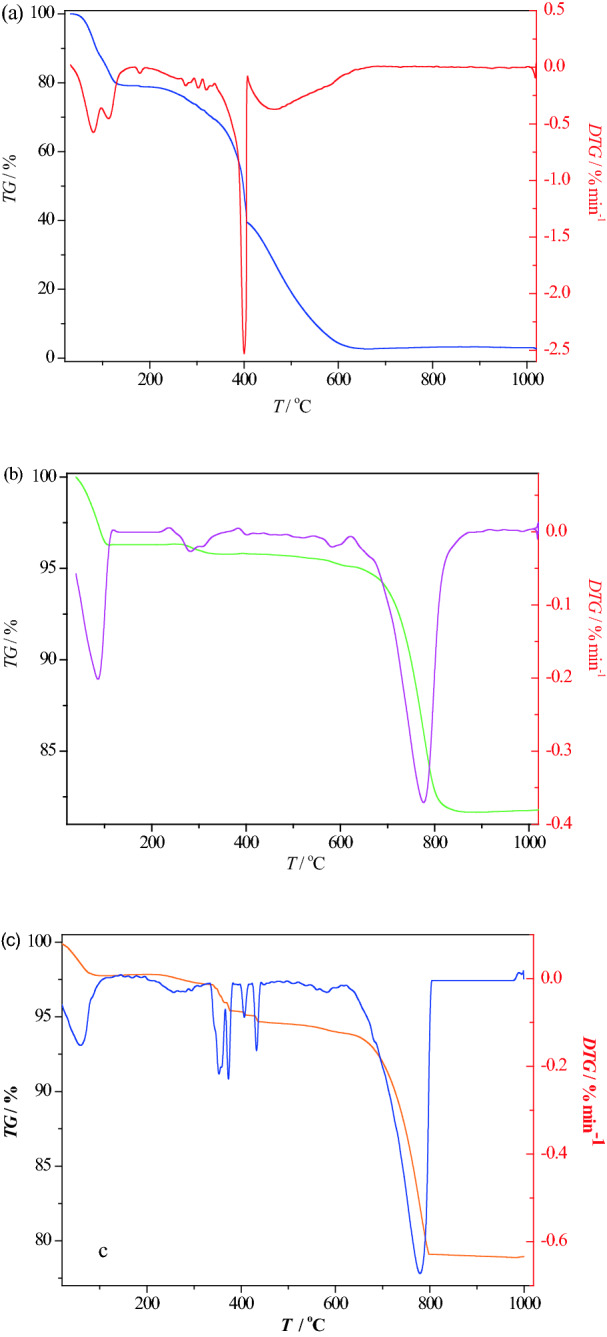
TG/DTG curves of methylene blue (**a**), NiO/sepiolite (**b**) and NiO/sepiolite adsorbed with methylene blue (**c**) under 20 cm^3^/min of O_2_ (heating rate = 10 °C/min).

As a thermal analysis (TA) technique, thermogravimetry/derivative thermogravimetry (TG/DTG) has been used to investigate the thermal behavior of compounds and the effect of a catalyst on the catalytic degradation of pollutants. Figure 4a–c shows the TG/DTG curves of methylene blue, NiO/sepiolite and NiO/sepiolite adsorbed with methylene blue, respectively. The removal of adsorbed water from the external surface of the methylene blue was completed up to 100 °C with a 15% weight loss (Fig. 4a). When increasing the temperature from 100 to 400 °C, a weight loss of approximately 50% was observed. This was ascribed to the combustion of organic matter, and it showed a significant decomposition at 400 °C. Then the decomposition process ended at 600 °C. Figure 4b shows the TG/DTG curves of the NiO/sepiolite. The adsorbed water on the surface of the sepiolite was removed at 100 °C with a 3% weight loss. The first portion of the bound water left the structure at 250–300 °C, with a weight loss of 1%^37^. The zeolitic water in the channel-type voids of the structure was not found because the NiO/sepiolite was calcined at 350 °C during sample preparation. The second portion of the bound water began to leave the structure at a higher temperature. The remaining bound water was removed at 750 °C and was accompanied by a 14% additional weight loss. Figure 4c shows the TG/DTG curves of the NiO/sepiolite adsorbed with the methylene blue. This TG/DTG curve shows an obvious 2% weight loss at 350 °C ascribed to the decomposition of the methylene blue. The degradation temperature was significantly changed during the presence of the catalyst, lowering the degradation temperature from 400 to 350 °C. It was clear that the modified sepiolite was useful to induce the oxidation of organic matter. Salvador et al. also reported that NiOx-modified sepiolite could effectively induce dehydrohalogenation and oxidation of a chlorinated pesticide (Lindane)^38^.

### Adsorption

**Figure 5:**
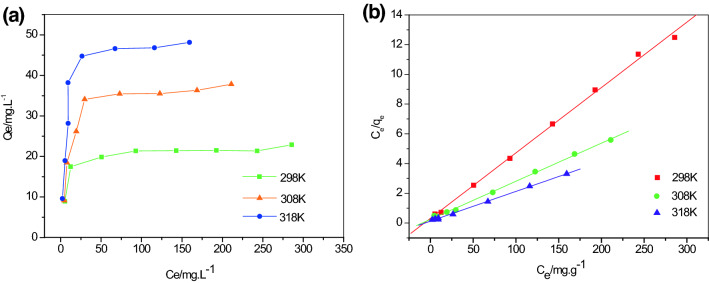
Adsorption isotherms NiO/sepiolite (**a**), isothermal adsorption of NiO/sepiolite: filled circles represent experimental data and the line represents the simulated Langmuir model (**b**).

The adsorption capacity and temperature are the most important characteristics in the determination of the adsorption efficiency of NiO/sepiolite. Typically, during the liquid phase, adsorption is endothermic. Increasing the temperature can increase the rate of diffusion of adsorbate molecules across the external boundary layer and in the internal pores of the adsorbent particle, causing the viscosity of the solution to decrease^39^. Adsorption isotherms of the NiO/sepiolite were examined at 298 K, 308 K, and 318 K, and the corresponding results are displayed in Fig. 5a. The results showed that the temperature had a great influence on the adsorption capacity, which increased with an increase in the temperature, and it was an endothermic process. Langmuir models were used to attempt to fit isotherms, and the results are presented in Fig. 5b. The correlation coefficients at the temperatures of 298, 308, and 318 K were 0.9977, 0.9981, and 0.9984, respectively, suggesting that the adsorption isotherm data followed the Langmuir model. Based on the Langmuir isotherm, the maximal absorption capacities of the NiO/sepiolite materials were 50, 39.06, and 22.73 mg/g at the temperatures of 318, 308, and 298 K, respectively. The adsorption isotherm study indicated that the NiO/sepiolite exhibited a high adsorption potential for the removal of methylene blue from an aqueous solution. These results show that temperature has a great influence on adsorption capacity, which increases with the increase of temperature, it is an endothermic process. Freundlich and Langmuir models are used to fit isotherms. The results of Freundlich and Langmuir models analyses suggest that adsorption isotherm data follow the Langmuir model (data about Freundlich model fitting is not showed). The fitting results are presented in Fig. 5b and Table 2. The adsorption isotherm study indicates that NiO/sepiolite exhibits a high adsorption potential for the removal of methylene blue from aqueous solution.

**Table 2:** Langmuir parameters and correlation coefficients for methylene blue adsorption onto NiO/sepiolite.

Temperature/K	Langmuir isotherm $\frac{{C_{e} }}{{q_{e} }} = \frac{{C_{e} }}{{q_{m} }} + \frac{1}{{q_{m} K_{L} }}$		
	q_m_ (mg g^−1^)	K_L_ (dm^3^ mg^−1^)	R^2^
298	22.73	0.1388	0.9977
308	39.06	0.1047	0.9981
318	50.00	0.1609	0.9984

### Regeneration

**Figure 6:**
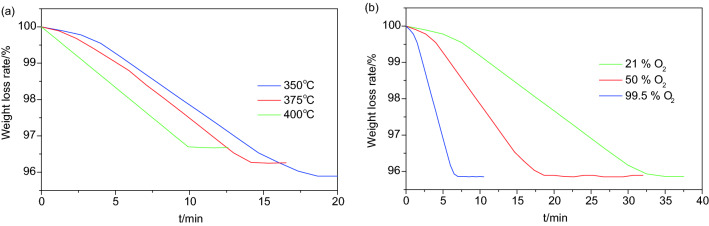
TG curves obtained in the degradation of methylene blue adsorbed on NiO/sepiolite at different temperature (**a**). TG curves obtained in the degradation of methylene blue adsorbed on NiO/sepiolite at different volume fraction of O_2_ (**b**).

The NiO/sepiolite was regenerated by high-temperature gas flow, and the effect of the regeneration temperature and the volume fraction of O_2_ on the regeneration rate were studied. Temperature is one of the most important factors in high-temperature gas flow regeneration. The effect of the regeneration temperature on the regeneration rate was studied using a simultaneous DTA/TG system. Methylene blue adsorbed on the NiO/sepiolite was oxidized by oxygen with volume fraction of 50% and a gas flow rate of 6 L/min at different temperatures (350 °C, 375 °C, and 400 °C). When the temperature rose to the set value, it was kept constant to record the TG data. The TG curves obtained from the degradation of methylene blue adsorbed on the NiO/sepiolite at different temperatures are shown in Fig. 6a. As expected, the order of the weight loss rates was 400 °C > 375 °C > 350 °C. It is apparent that higher temperatures were beneficial for the degradation of dye and regeneration of NiO/sepiolite. At 350 °C, it was observed that catalysts adsorbed with the dye showed no more weightlessness until 18 min, and the mass loss (4%) was in a good agreement with the saturated adsorption amount of methylene blue on the NiO/sepiolite (39.06 mg/g). The mass loss was due to the complete degradation of the dye. In addition, the catalysts achieved complete regeneration. At 375 °C, the removal of dye was completed at 14 min, with a mass loss of approximately 3.47%. With a higher degradation temperature at 400 °C, the complete removal of the dye was completed at 9 min. This was accompanied by an approximate 3.3% weight loss. At 400 °C, the weight loss had no change compared with that of the two other lower temperatures with relatively short time periods. It is noteworthy that the weight loss decreased with an increase in the temperature due to the degradation of more dye when the temperature increased from 350 to 400 °C. Hence, the regeneration of NiO/sepiolite was favored at higher temperatures.

The effect of the volume fraction of O_2_ on the regeneration rate was also studied using the simultaneous DTA/TG system. The methylene blue that adsorbed on the NiO/sepiolite was oxidized by oxygen with volume fractions of 21%, 50% and 99.5%, the gas flow rate was 6 L/min, and the temperature was maintained at 350 °C to record the TG data. The results are shown in Fig. 6b. It can be seen from Fig. 6b that the NiO/sepiolite catalysts required 7, 18 and 32.5 min to reach the end of regeneration, when the volume fractions of oxygen were 99.5%, 50% and 21%, respectively. These results illustrated that the regeneration efficiency was strongly oxygen concentration-dependent, the oxidation ability of O_2_ was related to its volume fraction, and it showed a stronger oxidation ability by increasing the volume fraction accordingly, and these factors favored the regeneration of the NiO/sepiolite.

In summary, the thermal studies were significantly helpful to understand the thermal behavior of the compounds. In addition, they illuminated the influences of the regeneration temperature and volume fraction of O_2_ on the regeneration rate. The degradation rate of the dye and the regeneration of catalysts were strongly dependent on the process temperature and volume fraction of O_2_.

**Figure 7:**
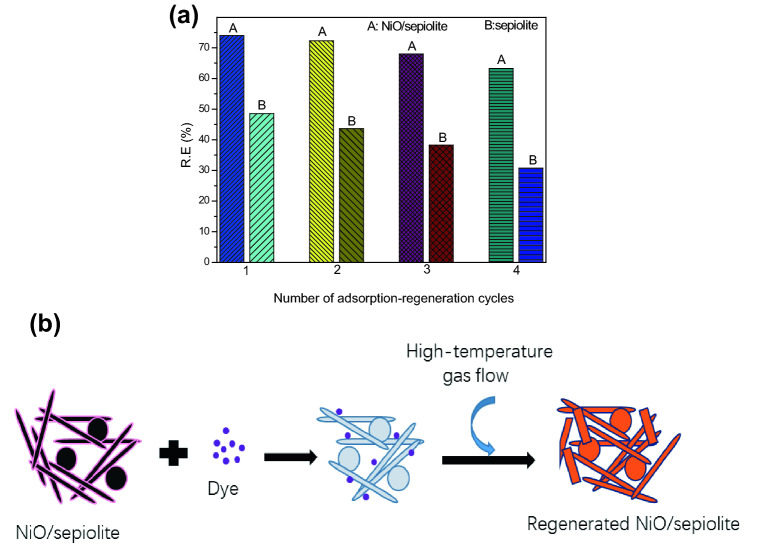
Regeneration rate over four cycles of adsorption and regeneration for removal of dye from aqueous solution using NiO/sepiolite and sepiolite (**a**), Schematic diagram of the adsorption and high-temperature gas flow regeneration process on NiO/sepiolite (**b**).

We also studied the stability of NiO/sepiolite. Figure 7a shows the regeneration rate over four cycles of adsorption and high-temperature gas flow regeneration for removing dye using NiO/sepiolite or natural sepiolite. NiO/sepiolite was found to be sufficient for achieving a 74% regeneration rate, delivering a significantly higher regeneration rate compared to that of natural sepiolite at 48.5%. In the case of NiO/sepiolite, the regeneration rate was still nearly the same as the first time, which was attributed to its modification treatment. After four circulations, the regeneration rate of NiO/sepiolite was 63.26%, while that of raw sepiolite was 30.76%. This indicated that the catalytic activity of NiO/sepiolite was much higher than that of natural sepiolite. The process of adsorption of dye on NiO/sepiolite and its regeneration by high-temperature gas flow is showed in Fig. 7b, first, the dye was adsorbed on the surface of NiO/sepiolite, then the NiO/sepiolite adsorbed with dye was regenerated by high-temperature gas flow and reused.

### Reaction kinetics

The reaction kinetics was obtained by a series of experiments. First, we determine the reaction order. In the initial stage of the regeneration process of NiO/sepiolite under O_2_ atmosphere, the reaction gas O_2_ is uniformly distributed inside the particles, which can be described by a uniform reaction model^40–42^. The regeneration reaction order is obtained by the average reaction rate under different O_2_ volume fractions. The uniform reaction model is expressed as the following Eq. (2)^42^:

2 $$\frac{{{\text{d}}X}}{{{\text{d}}t}} = k(1 - X)C_{{O_{2} }}^{n}$$

where X is the regeneration rate of NiO/sepiolite, t is time (s), k is the reaction rate constant, ${\text{C}}_{{{\text{O}}_{{2}} }}$ is the concentration of O_2_ (mol cm^−3^), and n is the reaction order. Woods et al. also used this expression to obtain the kinetic equation of ZnO–TiO_2_ adsorbents^43,44^. This uniform reaction model expression can be transformed into the following Eq. (3) by logarithm:

3 $$\frac{{{\text{d}}X}}{{{\text{d}}t(1 - X)}} = \ln k + n\ln C_{{O_{2} }}^{{}}$$

**Figure 8:**
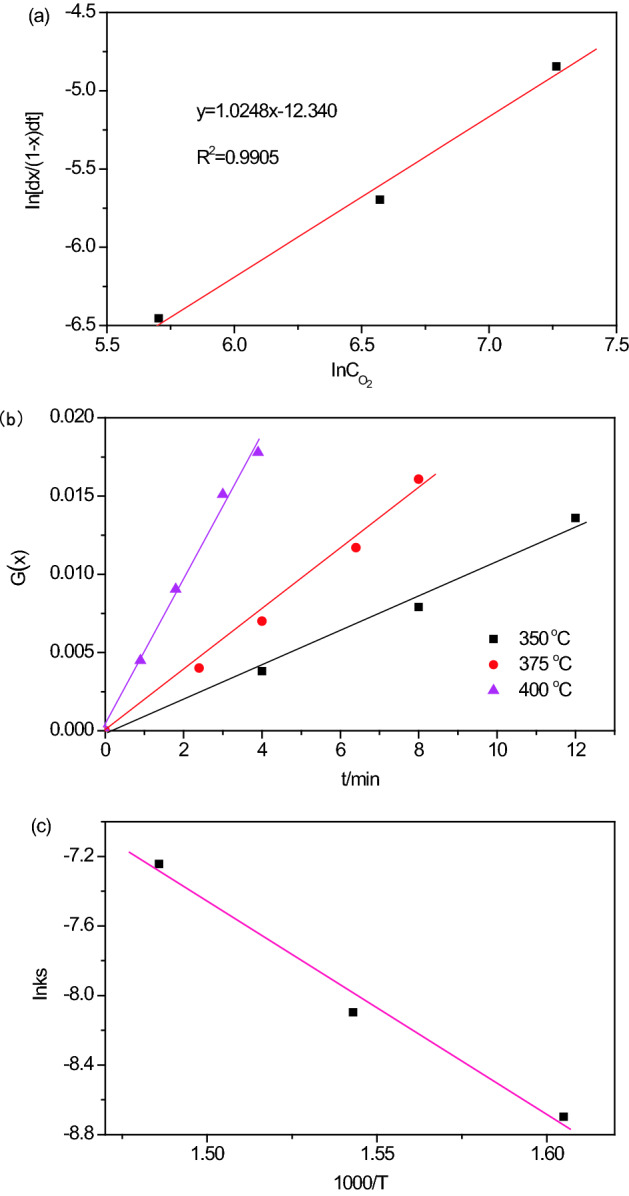
The relationship between the regeneration rate of NiO/sepiolite and O_2_ content (**a**), relationship between G(x) and t (**b**), relationship between lnk and 1/T (**c**).

The reaction order n can be determined by the TGA data from 350 to 400 °C (Fig. 6a,b), and we assume that the order of O_2_ in regeneration reaction to be 1, that is, n = 1, this value is substituted into Eq. (3), and calculate three different points according to Fig. 6a,b, then drawing the relationship between the regeneration rate of NiO/sepiolite and O_2_ content, the results are showed in Fig. 8a. We can see that the slope (n) is 1.0248, the correlation coefficient (R) is 0.9905, the results show that the assumption of reaction order n is reasonable, therefore, the reaction order of regeneration under O_2_ is a first-order reaction, the kinetic equation can be expressed as the following Eq. (4):

4 $$- r = \frac{{{\text{d}}X}}{{{\text{d}}t}} = k(1 - X)C_{{O_{2} }}$$

Second, we work out the apparent kinetics parameters. The kinetic behavior of the regeneration reaction could be described by the equivalent particle model. This model parameters are described as the following Eqs. (5), (6) and (7):

5 $$t = AG(X)$$

6 $$G(X) = 1 - (1 - X)^{1/2}$$

7 $$A = \rho_{o} R_{o} /kC_{{O_{2} }}$$

where G(x) is the characteristic function of surface reaction control, X is the regeneration rate, t is time (s), k is the reaction rate constants, R_o_ is the radius of particle (3 × 10^–3^ m), Co_2_ is the mass concentration of O_2_ (7.14 × 10^2^ g m^−3^), ρ_o_ is the grain density of dye (3.62 × 10^4^ g m^−3^). On the basis of data obtained at 50% O_2_ (volume fraction, reaction at 350, 375 and 400 °C), the values of G(x) under different regeneration rate are calculated according to Eqs. (5), (6) and (7), and the results are shown in Fig. 8b, the fitting results are listed in Table 3. From Table 3 we can see that the reaction rate constants k increase with the increase of regeneration temperature, this means that as the reaction temperature increases, the reaction rate also increases. All of the correlation coefficient R^2^ are above 0.99.

**Table 3:** The curve fitting of G(x)-t and relevant parameters.

Temperature (°C)	G(x)-t fitting equation	k	R^2^
350	G(x) = 0.0011t − 0.0005	1.67 × 10^–4^	0.9949
375	G(x) = 0.0020t − 0.0005	3.04 × 10^–4^	0.9921
400	G(x) = 0.0047t − 0.0003	7.15 × 10^–4^	0.9937

After taking logarithm, the Arrhenius equation (Eq. 8) can be transformed into Eq. (9).

8 $$k = A\exp ( - E_{a} /RT)$$

9 $$\ln k = - \frac{{E_{a} }}{RT} + \ln A$$

where A is the pre-exponential factor, *k* is the reaction rate constant, Ea is the activation energy (kJ mol^−1^), R is the gas constant (8.314 J mol^−1^ K^−1^), T is the absolute temperature (T). When plotting of lnk with 1/T (showed in Fig. 8c), the fitting equation is lnk_s_ = − 12.172 × (1000/T) + 10.789, the pre-exponential factor A is 4.85 × 10^4^ m s^−1^, the activation energy Ea is 101.20 kJ mol^−1^, which indicates that the regeneration reactivity is quite sensitive to temperature^42^. Therefore, the Eq. (4) can be transformed into Eq. (10), which is the apparent kinetic reaction of regeneration reaction under high-temperature gas flow.

10 $$- r = \exp \left( {\frac{{12.172 \times 10^{3} }}{T} + 10.789} \right)(1 - X)C_{{O_{2} }}$$

## Conclusions

In summary, NiO/sepiolite materials were prepared using impregnation, and this was found to be an efficient adsorbent and active catalyst for removing dye in an adsorption-high-temperature gas regeneration single unit. The degradation temperature of the dye observed in the presence of the NiO/sepiolite was much lower than that with no catalyst. This catalyst had a high regeneration rate of 74% within only 7 min under an O_2_ gas flow. The reusability of the catalyst after regeneration was then investigated. The degradation rate of the dye adsorbed on the NiO/sepiolite and/or the regeneration of the catalysts were strongly influenced by the regeneration temperature and the volume fraction of O_2_. The reaction kinetic experiments demonstrated that the reaction order was 1. These adsorption and regeneration processes successfully eliminated organic pollutants. This study demonstrated the effectiveness of high-temperature gas degradation for dye, providing an efficient approach for the treatment of wastewater and regeneration of adsorbents and catalysts.

## Supplementary Information

Supplementary Information.
