# Supplementary material for: Recyclable NiO/sepiolite as adsorbent to remove organic dye and its regeneration
Source: Sci Rep. 2022 Feb 21;12:2895. doi: 10.1038/s41598-022-06849-6 (PMC8861065; doi:10.1038/s41598-022-06849-6)
Supplement: Supplementary file 1 — Supplementary Information. [file 41598_2022_6849_MOESM1_ESM.docx]

Schematic 1 (Supplementary figure)


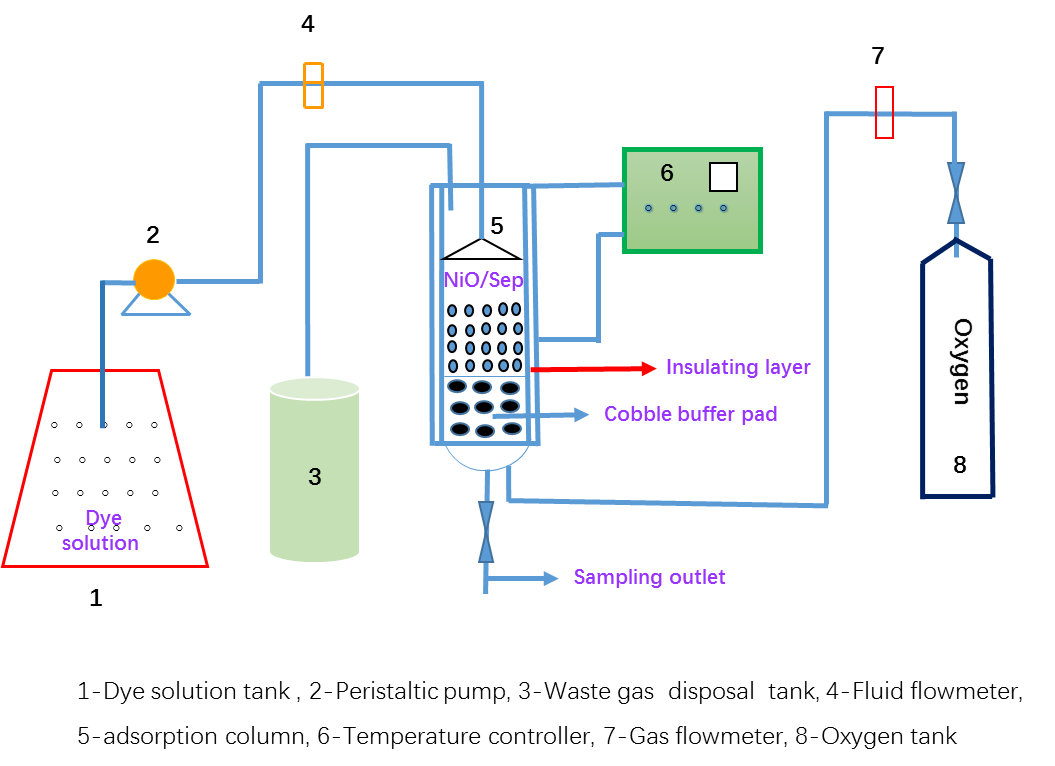


Schematic 1 The schematic of adsorption-regeneration cycle system
